# Variation in secondary metabolite production as well as antioxidant and antibacterial activities of *Zingiber zerumbet* (L.) at different stages of growth

**DOI:** 10.1186/s12906-016-1072-6

**Published:** 2016-03-22

**Authors:** Ali Ghasemzadeh, Hawa Z. E. Jaafar, Sadegh Ashkani, Asmah Rahmat, Abdul Shukor Juraimi, Adam Puteh, Mahmud Tengku Muda Mohamed

**Affiliations:** Department of Crop Science, Faculty of Agriculture, Universiti Putra Malaysia, 43400 Serdang, Selangor Malaysia; Institute of Tropical Agriculture, Universiti Putra Malaysia, 43400 Serdang, Selangor Malaysia; Department of Agronomy and Plant Breeding, Shahr-e- Rey Branch, Islamic Azad University, Tehran, Iran; Department of Nutrition & Dietetics, Faculty of Medicine & Health Sciences, Universiti Putra Malaysia, 43400 UPM Serdang, Selangor Malaysia

**Keywords:** *Zingiber zerumbet* (L.), Antibacterial, Chalcone synthase enzyme, DPPH, FRAP, Flavonoids, Phenolic acids

## Abstract

**Background:**

*Zingiber zerumbet* (L.) is a traditional Malaysian folk remedy that contains several interesting bioactive compounds of pharmaceutical quality.

**Methods:**

Total flavonoids and total phenolics content from the leaf, stem, and rhizome of *Z. zerumbet* at 3 different growth stages (3, 6, and 9 months) were determined using spectrophotometric methods and individual flavonoid and phenolic compounds were identified using ultra-high performance liquid chromatography method. Chalcone Synthase (CHS) activity was measured using a CHS assay. Antioxidant activities were evaluated by ferric reducing antioxidant potential (FRAP) and 1,1-diphenyl-2-picrylhydrazyl (DPPH) assays. The antibacterial activity was determined against Gram-positive and Gram-negative bacteria using the disc diffusion method.

**Results:**

Highest content of total flavonoid [29.7 mg quercetin equivalents (QE)/g dry material (DM)] and total phenolic (44.8 mg gallic acid equivalents (GAE)/g DM) were detected in the rhizome extracts of 9-month-old plants. As the plant matured from 3 to 9 months, the total flavonoid content (TFC) and total phenolic content (TPC) decreased in the leaf, but increased significantly in the rhizomes. Among the secondary metabolites identified, the most abundant, based on the concentrations, were as follows: flavonoids, catechin > quercetin > rutin > luteolin > myricetin > kaempferol; phenolic acids, gallic acid > ferulic acid > caffeic acid > cinnamic acid. Rhizome extracts from 9-month-old plants demonstrated the highest CHS activity (7.48 nkat/mg protein), followed by the 6-month-old rhizomes (5.79 nkat/mg protein) and 3-month-old leaf (4.76 nkat/mg protein). Nine-month-old rhizomes exhibited the highest DPPH activity (76.42 %), followed by the 6-month-old rhizomes (59.41 %) and 3-month-old leaves (57.82 %), with half maximal inhibitory concentration (IC_50_) of 55.8, 86.4, and 98.5 μg/mL, respectively, compared to that of α- tocopherol (84.19 %; 44.8 μg/mL) and butylated hydroxytoluene (BHT) (70.25 %; 58.6 μg/mL). The highest FRAP activity was observed in 9-month-old rhizomes, with IC_50_ of 62.4 μg/mL. Minimal Inhibitory Concentration (MIC) of *Z. zerumbet* extracts against Gram-positive and Gram-negative bacteria ranged from 30 to >100 µg/mL. Among the bacterial strains examined, *Staphylococcus aureus* was sensitive to the leaf extract of *Z. zerumbet,* with MIC of 30.0 μg/mL and other strains were sensitive to the rhizome extracts.

**Conclusions:**

Three- and 9-month-old plants are recommended when harvesting the leaf and rhizome of *Z. zerumbet*, respectively, in order to obtain effective pharmaceutical quality of the desired compounds*.*

## Background

Plants with medicinal potentials and their secondary metabolites have been identified and applicated in dishes from the earliest annals of human habitancy. The practices of plant-based traditional medicine are founded on hundreds of years of belief and observations, which predate the development of modern medicine. Herbs are a rich source of bioactive compounds, such as phenolic acid and flavonoid, and are used as pharmaceutical intermediates and chemical entities for the drug synthesis [1]. Flavonoids and phenolics are essential groups of phytochemicals, which have demonstrated superoxide radical scavenging activity, and therefore exhibit anticancer properties [2, 3]. Dietary phenolics have recently attracted significant interest, owing to their antioxidant and possibly anti-carcinogenic activities [4–6]. Flavonoids are common constituents of plants used in traditional medicine to treat a wide range of diseases [7, 8]. Chalcone synthases, is a member of the plant polyketide synthase superfamily, provide the starting materials for flavonoids production in plants [9]. In fact, production of secondary metabolites (flavonoids and phenolics) in herbs is related to CHS activity which is strongly influenced by several parameters such as environmental conditions (light intensity, temperature), nutrient, stress and plant age [1, 10]. Herbal products are prepared from various parts of the plant (including the leaves, stems, trunks, roots, flowers, fruits, twigs, and seeds). To ensure the quality of the raw material, careful preparation procedures are followed: harvesting (herbs should be harvested at the optimum growth stage, when their qualities are at its peak), cleaning, drying, sizing, and storage. Furthermore, a number of factors, such as environmental and cultivation conditions, management practices (temperature, irradiance, fertilizer supply, and irrigation), and the age of the plant (harvesting time) have been considered to significantly affect the level of bioactive compounds in herbs and crops [11, 12]. *Zingiber zerumbet* (L.) Roscoe ex Sm., locally known as “lempoyang” in Malaysia and “shampoo ginger” in English, is a plant that belongs to the Zingiberaceae family, under which the ginger species are categorized. *Z. zerumbet* is a perennial, tuberous root plant that grows naturally in the damp and shaded parts of the lowland or hill slopes, as scattered plants or thickets. This herbal plant is believed to be native to India and the Malaysian Peninsula [13]. In Malaysia, *Z. zerumbet* is one of the traditional folk remedies that contain several interesting bioactive compounds with anti-tumor [14], antioxidant [13], anti-pyretic and analgesic activity [15], antibacterial activity [16] anti-inflammatory, anti-alergic activity [17] and anti-hypersensitive activity [18]. Due to the wide range of beneficial effects of this herb, studies are necessary to investigate the alteration in the production of phytochemicals throughout the plant organs, in relation to the age of the plant. It is important to gather relevant evidence regarding herbs with high levels of potentially beneficial components. A recent study conducted by Chien et al. [19] reported that the rhizomes from 8-month-old *Z. zerumbet* plants demonstrated the strongest anti-inflammatory activity, compared to the maximum activity of zerumbone. Currently, the production of secondary metabolites and the variation in the levels of bioactive components during the maturation of *Z. zerumbet* remain largely unexplored. The objective of the present study was to evaluate the phytochemical production in different parts of the *Z. zerumbet* plant, in relation to chalcone synthase enzyme activity, and determine their antioxidant and antibacterial activities at different stages of maturity.

## Methods

### Plant sampling

The rhizomes of *Z. zerumbet* were soaked in mancozeb solution (0.3 %) for 30 min. The rhizomes were cut into sections (3–5 cm) and contained two to three buds. The rhizomes were germinated for two weeks in growing pots (15 × 15 cm) filled with peat moss (each weighing approximately 1 kg). The rhizomes were planted 6 cm deep into peat moss with the buds facing upward. The rhizomes were germinated under glasshouse condition. After 2 weeks, when the young leaves of the seedlings reached 5 cm in height, the seedlings were transplanted into polyethylene bag (45 × 38 cm) and filled with a soilless mixture, which contained burnt rice husk and coco peat at a 1 : 1 ratio (each weighing approximately 6 kg). Water was supplied to individual plants (1.2 L/day) by screwtype nozzles. Fertilizer in recommended dose [20] was applied efficiently through the drip system. The experiment was conducted at the glasshouse complex of the University Putra Malaysia (UPM). The mean daily temperature was 30 °C, mean relative humidity was 70–80 %, and the highest and lowest irradiance levels were 1650 μmol/m^2^/s and 44 μmol/m^2^/s, respectively. Plants were harvested at 3, 6, and 9 months after plantation (seedling, young and mature stages). Rhizomes, leaves, and stems were separated and washed with pure water. Separated parts of the plant were freeze-dried and kept at −20 °C. Samples were submitted to Institute of Bioscience (University Putra Malaysia) and were identified as *Z. zerumbet* with voucher specimen of SK622/07. Voucher specimens deposited at herbarium of Institute of Bioscience, University Putra Malaysia.

### Extraction

Dried samples (50 g) were grounded into powder followed by extraction with distilled water (1 L). Solutions were refluxed for 2 h at 65 °C, then cooled and filtered through Whatman filter paper (No. 1) in a filter funnel, followed by evaporation under reduced pressure in an Eyela rotary evaporator to remove excess solvent. Residue was freeze dried and dried extracts were kept at −20 °C for future analysis.

### Total phenolic content

The total phenolic concentration in the extracts of different parts of plant was determined as described by Jayaprakasha and Patil [21], with some modification. Crude extract of leaf, stem and rhizome (0.25 mg) was dissolved in methanol (10 mL) and 200 μL of solution were diluted in 20 mL of distilled water. Folin-Ciocalteu reagent (10-fold diluted; 1 mL) was added and the mixture was incubated in total darkness for 10 min at room temperature. After this time, sodium carbonate 7.5 % (1 mL) was added and incubated for 30 min, then the absorbance of the solution was read at 765 nm using a spectrophotometer (UV2550, Shimadzu, Japan). Different concentrations of gallic acid were used to prepare a calibration curve. Results were expressed as milligram gallic acid equivalents (GAE)/g DM. Measurement was performed in triplicate and values are the average of three replicates.

### Total flavonoid content

Crude extract of leaf, stem and rhizome (0.25 mg) was dissolved in methanol (10 mL). Extracts of leaf, stem and rhizome (1 mL) were mixed with NaNO_2_ solution (4 mL, 1:5, w/v) and incubated at room temperature for 6 min. After this time, 0.3 mL of AlCl_3_ solution (1:10, w/v) was added, the reagents were mixed well, and the reaction was allowed to stand for another 6 min. Immediately after that, 1 M NaOH solution (2.0 mL) was added to each extract and incubated for 10 min at room temperature. The absorbance of the solutions was read at 510 nm using a spectrophotometer (UV2550, Shimadzu, Japan). Different concentrations of quercetin standard were used to prepare a calibration curve [1]. Results were expressed as milligram quercetin equivalents (QE)/g DM. Measurement was performed in triplicate and values are the average of three replicates.

### Separation and analysis of flavonoids and phenolic acids

Ultra-high performance liquid chromatography (UHPLC, 1290 Infinity Quaternary LC System, Agilent, Santa Clara, CA, USA) was used to separate and identify the phenolic acids. The chromatographic system conditions were set as follows: mobile phase, 0.03 M orthophosphoric acid (A) and methanol HPLC grade (B); detector, UV 360 nm; column, C18 column (5.0 μm, 4.6 mm inner diameter [ID] × 250 mm); column oven temperature, 35 °C; and flow rate, 1.0 mL/min. Gradient elution was performed as follows: 0–10 min, 10 % B; 10–15 min, 50 % B; 15–20 min, 100 % B; and finally 5 min for washing. All standards (catechin, quercetin dihydrate, rutin hydrate, luteolin, myricetin, kaempferol, gallic acid, ferulic acid, trans-caffeic acid and trans-cinnamic acid were purchased from Sigma-Aldrich (M) Sdn Bhd, Selangor, Malaysia) were dissolved in methanol HPLC grade. Linear regression equations were calculated using Y = aX ± b, where X is the concentration of the related compound and Y the peak area of the compound obtained from UHPLC. The linearity was established by the coefficient of determination (R^2^). UHPLC analysis was performed in triplicate and values are the average of three replicates.

### Chalcone Synthase (CHS) assay

The CHS was extracted from 0.4 g of plant samples with a solution of 1 mM 2-mercaptoethanol dissolved in 0.1 M borate buffer (1 mL, pH 8.8) at 4 °C. Subsequently, Dowex l × 4 resin (0.1 g) was added to the solution and the mixture rested for 10 min. The solution was then centrifuged at 15,000 rpm for 10 min to remove the resin. The supernatant was transferred to a tube, and Dowex resin (0.2 g) was added and the mixture left standing for 20 min. The resin was removed from solution after centrifugation at 15,000 rpm for 15 min. The supernatant (100 μL) was mixed gently with 10 mM potassium cyanide and following that Tris-HCI buffer (1.89 mL, pH 7.8) was added. Subsequently, chalcone (10 mg) was added to ethylene glycol monomethyl ether (10 μL), mixed with enzyme extract, and the reaction allowed to proceed for 1 min at 30 °C. The absorbance was measured at 370 nm using spectrophotometer (UV2550, Shimadzu, Japan).

### In vitro evaluation of antioxidant activity

#### 1,1-Diphenyl-2-picrylhydrazyl (DPPH) assay

The DPPH assay was used in order to evaluate the free radical scavenging activity of *Z. zerumbet* extracts. DPPH was dissolved in methanol at a concentration of 100 μM. The DPPH solution (3 mL) was mixed with 3 mL of various concentrations (10, 20, 40, 80 and 160 μg/mL) of *Z. zerumbet* extracts and incubated in a dark room for 20 min at 27 °C. After incubation, the absorbance of the samples was read at 517 nm. Butylated hydroxytoluene (BHT) and α-tocopherol were used as a positive control [22]. The scavenging activity was calculated using the following formula:

1 $$ \%\ \mathrm{inhibition} = \left[\left({\mathrm{absorbance}}_{\mathrm{control}}\hbox{--}\ {\mathrm{absorbance}}_{\mathrm{sample}}\right)/{\mathrm{absorbance}}_{\mathrm{control}}\right)\Big] \times 100 $$

#### Ferric Reducing Antioxidant Potential (FRAP) assay

The stock solutions consisted of 10 volume of 300 mM acetate buffer (PH = 3.6), 1 volume of 10 mM TPTZ (2,4,6-tripyridyl-S-triazine) solution in 40 mM HCl, and I volume of 20 mM FeCl_3_ solution. Acetate buffer (25 mL) and TPTZ (2.5 mL) were mixed (FRAP solution), and 2.5 mL FeCl_3_ added. Plant extracts (100 μL) and deionized water (300 μL) was added to 3 mL of the FRAP solution and incubated for 30 min at 37 ˚C in the dark water bath. The absorbance of the resultant solution was measured at 593 nm. Acetate buffer was used as a blank reading. A standard curve was prepared using various concentrations of FeSO_4_ × 7H_2_O. The difference between sample absorbance and blank absorbance was calculated and used to calculate the FRAP value [23].

### Antibacterial activity

#### Preparation of plant extracts

Fifty grams of dried samples was extracted with 200 mL ethanol using a soxhlet evaporator for 48 h. After complete solvent evaporation, 50 mg of extracts were dissolved in 1 mL of 10 % dimethyl sulphoxide (DMSO) (Merck, Malaysia) to a final concentration of 50 mg/mL and stored at 5 °C for further use.

#### Bacterial cultures and growth conditions

MDR clinical isolates of Gram positive bacterium (*Staphylococcus aureus, Bacillus subtilis, Listeria monocytogenes*) and Gram negative bacterium (*Escherichia coli, Salmonella typhimurium* and *Pseudomonas aeruginosa*) with their antibiotic resistance profiles were obtained from the laboratory of microbial culture collection, Institute Bio-sience, University Putra Malaysia, Selangor, Malaysia. All the test strains were maintained on nutrient agar slants at 4° and sub-cultured on to nutrient broth for 24 h prior to testing. These bacteria served as test pathogens for antibacterial activity assay.

#### Antibacterial activity assay

Fifteen milliliters of the molten agar (45 °C) were poured into sterile Petri dishes (90 mm). Cell suspensions were prepared and 100 μL was evenly distributed onto the surface of the agar plates of Mueller-Hinton agar. Once the plates had been aseptically dried, 6 mm wells were punched into the agar with a sterile Pasteur pipette. The different extracts (50 mg/mL) were diluted with dimethylsulfoxide (DMSO): water (1:9) to give concentration of 10 mg/mL and 80 μL of diluted extracts were placed into the wells and the plates were incubated at 37 °C for 24 h. Gentamicin and ciprofloxacin (25 μL/wells at concentration of 4 μg/mL) were used as positive control for bacteria. Antibacterial activity was evaluated by measuring the diameter of circular inhibition zones around the well. Tests were performed in triplicate and values are the averages of three replicates. Data were expressed as mean ± standard deviation.

#### Minimum Inhibitory Concentration (MIC)

Based on the previous screening the minimum inhibitory concentration (MIC) of plant extracts was analyzed through the agar-well diffusion method. A bacterial suspension (10^5^–10^6^ CFU/mL) of each tested microorganism was spread on the nutrient agar plate. The wells (6 mm diameter) were cut from agar, and 60 μL of each plant extracts dissolved in dimethyl sulfoxide (DMSO): water (1:9) at different concentrations (10–100 μg/mL) were delivered into them. The plates were incubated at 37 °C for 24 h under aerobic conditions. After incubation, diameter of the inhibition zone was measured. MIC was taken from the concentration of the lowest dosed well visually under light microscope (Y100, USA) showing no growth after 24 h. All samples were tested in triplicates.

### Statistical analysis

All data were analyzed using analysis of variance by Statistical Analysis System (SAS version 9.2, SAS Institute Inc.). Mean separation test between treatments was performed using Duncanʼs Multiple Range Test and P-value of < 0.05 was regarded as significant.

## Results and discussion

### Total flavonoid content and identification of flavonoid compounds

Table 1 shows the total flavonoid content (TFC) and the identified flavonoid compounds from leaf, stem and rhizome extracts of *Z. zerumbet *. TFC was significantly affected by the age of the plant and varied in different plant parts. Nine-month-old whole plant samples (leaf, stem, and rhizome) showed the highest TFC (49 mg QE/g DM) followed by the 6-month-old (42.9 mg QE/g DM) and 3-month-old (37.7 mg QE/g DM) plants. As the age of the plant increased from 3 to 9 months, enhanced TFC was detected throughout the plants. Among the parts of the plant investigated, the highest TFC (29.7 mg QE/g DM) was observed in the rhizomes of the 9-month-old plants. The most striking result was that during plant maturity from 3 to 9 months, TFC of the leaf decreased significantly from 21.8 to 15.2 mg QE/g DM. On the contrary, rhizomes showed different results to those of the leaf. As the plant matured from 3 to 9 months, TFC increased significantly in the rhizomes, from 11.2 to 29.7 mg QE/g DM. The TFC in the stems did not differ significantly among the different growth stages. In the present study, 6 flavonoid compounds, including quercetin, rutin, kaempferol, catechin, luteolin, and myricetin were identified. Quercetin is a flavonoid found in several herbs and has shown potent anticancer and antioxidant activity [24]. Different concentrations of quercetin have been reported in different herbs. Levels of quercetin in *Z. zerumbet* varied in different plant age and parts. The highest content of quercetin (3.94 mg/g DM) was observed in the rhizome extracts obtained from the 9-month-old plant, followed by the  6-month-old rhizomes (2.49 mg/g DM) and leaf extracts (2.43 mg/g DM) from 3-month-old plants. Quercetin content in *Z. zerumbet*, when compared with other plants, for example red chili (0.799 mg/g DM), bird's eye chili (0.392 mg/g DM), bell pepper (0.448 mg/g DM), black tea (1.107 mg/g DM), onion (1.49 mg/g DM) [25] and ginger rhizome (0.86 mg/g DM) [4] was markedly higher in the leaves and rhizomes. The results of the present study are inconsistent with the findings of Behn et al. [26], who reported that the lowest quercetin content was observed in the young leaves of red leaf lettuce compared to that observed in the older leaves. Further, rutin content was influenced by the age of the plant and differed in different parts of the plant. Similar to quercetin, the highest content of rutin (2.79 mg/g DM) was observed in the rhizome extract of 9-month-old plants, followed by 6-month-old rhizome (2.19 mg/g DM) and 3-month-old leaf (1.77 mg/g DM). Kaempferol is a rare flavonoid compound and in this study, the highest concentration of kaempferol (0.72 mg/g DM) was observed in the 9-month-old rhizome. However, differing from the concentrations reported for green chili (0.039 mg/g DM), white radish (0.0383 mg/g DM), asiatic pennywort (0.0205 mg/g DM) [25], and ginger (0.06 mg/g DM) [4]. *Z. zerumbet* showed high concentrations of kaempferol (0.12–0.72 mg/g DM). Kaempferol was not detected in the stem extracts of the 3 and 6-month-old plants. Catechin content was significantly affected by the age and parts of the plant. As the plant matured from 3 to 9 months, enhanced catechin content was observed in the rhizome (2.69 to 6.12 mg/g DM) but decreased in the leaves (5.49 to 3.37 mg/g DM). Luteolin, is a flavonoid commonly found in several types of fruits and vegetables but in varying concentrations. In this study, luteolin was detected in the leaf and rhizome extracts at concentrations of 0.44–1.15 mg/g DM. The highest concentration of luteolin was observed in the 3-month-old leaf, followed by the 9-month-old rhizome; however the concentration of luteolin did not differ significantly between the 3-month-old leaf 9-month-old rhizomes. Myricetin belongs to the flavonoid class of polyphenolic compounds, and has shown antioxidant properties. Highest content of myricetin was detected in the 9-month-old rhizome (1.19 mg/g DM), followed by the 6-month-old rhizome (0.97 mg/g DM). Kaempferol was not observed in the 3-month-old leaf or the 3- and 6-month-old stem extracts.

**Table 1:** Total flavonoid content and individual flavonoid compounds from leaf, stem and rhizome extracts of  *Z. zerumbet*

Plant age (month-old)	Plant parts	Total flavonoids	Quercetin	Rutin	Kaempferol	Catechin	Luteolin	Myricetin
3	leaf	21.8 ± 3.74^b^	2.43 ± 0.69^b^	1.77 ± 0.26^c^	0.71 ± 0.09^a^	5.49 ± 0.45^b^	1.15 ± 0.14^a^	ND
	stem	4.7 ± 0.68^e^	0.12 ± 0.05^e^	0.36 ± 0.07^g^	ND	1.14 ± 0.10^h^	ND	ND
	rhizome	11.2 ± 1.26^d^	1.63 ± 0.41^c^	0.81 ± 0.09^e^	0.49 ± 0.04^b^	2.69 ± 0.29^f^	0.44 ± 0.03^c^	0.59 ± 0.03^c^
6	leaf	19.1 ± 2.69^b^	2.26 ± 0.52^b^	1.28 ± 0.32^d^	0.51 ± 0.04^b^	4.12 ± 0.21^d^	0.82 ± 0.06^b^	0.25 ± 0.02^e^
	stem	4.2 ± 0.65^e^	0.16 ± 0.05^e^	0.21 ± 0.04^h^	ND	1.34 ± 0.11^g^	ND	ND
	rhizome	19.6 ± 3.48^b^	2.49 ± 0.33^b^	2.19 ± 0.41^b^	0.68 ± 0.07^a^	4.68 ± 0.27^c^	0.76 ± 0.07^b^	0.97 ± 0.06^b^
9	leaf	15.2 ± 2.68^c^	1.82 ± 0.24^c^	0.66 ± 0.08^f^	0.50 ± 0.03^b^	3.37 ± 0.41^e^	0.45 ± 0.01^c^	0.33 ± 0.01^d^
	stem	4.1 ± 0.66^e^	0.22 ± 0.06^d^	0.24 ± 0.07^h^	0.12 ± 0.01^c^	1.02 ± 0.06^i^	ND	0.11 ± 0.02^f^
	rhizome	29.7 ± 4.11^a^	3.94 ± 0.81^a^	2.79 ± 0.48^a^	0.72 ± 0.05^a^	6.12 ± 0.32^a^	1.06 ± 0.11^a^	1.19 ± 0.11^a^

Data are means of triplicate measurements ± standard deviation. Means not sharing a common single letter in each column for each measurement were significantly different at P ≤ 0.05. The units of total flavonoids and flavonoid compounds are mg quercetin equivalents/g DM and mg/g DM, respectively. ND: not detected

Among the flavonoids identified in this study, the most abundant according to the highest concentrations were as follows: catechin > quercetin > rutin > luteolin > myricetin > kaempferol. Generally, during the plant maturity from 3 to 9 months old, the concentration of flavonoid decreased in the leaves (quercetin 25.1 %, rutin 62.7 %, kaempferol 29.5 %, catechin 38.6 %, and luteolin 60.8 %) and increased in the rhizomes (quercetin 141.7 %, rutin 244.4 %, kaempferol 46.9 %, catechin 127.5 %, luteolin 140.9 %, and myricetin 101.6 %). A study on ginger showed that the concentration of flavonoid compounds decreased significantly in the leaf, but increased in the rhizome during the growth of the plant [4]. evaluated the effect of harvesting time on the synthesis of flavonoid compounds in *Calendula officinalis,* and reported that the synthesis of 5 flavonoid compounds was influenced by the time of harvest. On the basis of the previous results and those of the current study, it is hypothesized that the duration of plant growth could have a significant impact on the synthesis of flavonoids in *Z. zerumbet*.

### Total phenolic content and identification of phenolic acid compounds

The results obtained from the preliminary analysis of phenolic compounds are shown in Table 2. TPC ranged from 5.9 to 44.8 mg GAE/g DM. TPC was significantly affected by the age of the plant and differed in different parts of the plant. In the leaf samples, the lowest (22.4 mg GAE/g DM) and highest (38.4 mg GAE/g DM) TPC were observed with the 9- and 3-month-old leaf, respectively. In the rhizome samples, the lowest (19.2 mg GAE/g DM) and highest (44.8 mg GAE/g DM) TPC were observed with the 3- and 9-month-old rhizomes, respectively. Compared to the TPC of the leaf and rhizomes, the stem samples demonstrated the lowest TPC (5.8–6.7 mg GAE/g DM). Further, the TPC did not differ significantly between the 3- and 6-month-old stems. In the present study, 4 phenolic compounds, including gallic acid, caffeic acid, ferulic acid, and cinnamic acid were determined in *Z. zerumbet* at three different stages of growth. Gallic acid was the most abundant (1.16 to 19.76 mg/g DM) among the phenolic acids identified in *Z. zerumbet* extracts. The highest and lowest concentrations of gallic acid were observed in the stem extracts of the 3-month-old plant and rhizome extracts of the 9-month-old plant, respectively. Caffeic acid was detected in *Z. zerumbet* extracts at concentration of 0.2–2.71 mg/g DM. The lowest and highest concentrations of caffeic acid were observed in the 3-month-old stem and 9-month-old rhizomes, respectively. Romani et al. [27] reported that the levels of caffeic acid was higher in the lettuce leaves at the earlier growth stage than at the later growth stages. In addition, ferulic acid was detected in *Z. zerumbet*. Interestingly, the highest concentration of ferulic acid (5.46 mg/g DM) was observed in the 3-month-old leaf extracts, followed by the 9-month-old rhizome (3.73 mg/g DM) and 6-month-old leaf (3.66 mg/g DM). Ferulic acid was not observed in the 3- and 6-month-old stems. Cinnamic acid and its derivatives are potent phenolic acids and have shown various biological effects for the treatment of several diseases [28]. In this study, cinnamic acid was detected in *Z. zerumbet* at concentration of 0.16–2.61 mg/g DM. The lowest and highest concentration of cinnamic acid were observed in the 3-month-old stem and 9-month-old rhizome, respectively. Among the phenolic acids identified, the most abundant according to the highest concentration were as follows: gallic acid > ferulic acid > caffeic acid > cinnamic acid. Similar to the results of the flavonoids, as plant age increased from 3 to 9 months, the content of phenolic acids decreased in the leaves (gallic acid 33.9 %, caffeic acid 28.0 %, ferulic acid 42.4 %, and cinnamic acid 42.0 %) and increased in the rhizomes (gallic acid 206.8 %, caffeic acid 243 %, ferulic acid 213.4 %, and cinnamic acid 137 %). Our results were consistent with previous studies and indicated that the synthesis and accumulation of phenolic compounds were affected by the age of the plant [1, 29, 30].

**Table 2:** Total phenolic content and individual phenolic compounds from leaf, stem and rhizome extracts of  *Z. zerumbet*

Plant age (month-old)	Plant parts	Total phenolics	Gallic acid	Caffeic acid	Ferulic acid	Cinnamic acid
3	leaf	38.4 ± 2.35^b^	11.62 ± 1.50^b^	1.46 ± 0.28^b^	5.46 ± 0.77^a^	1.88 ± 0.27^b^
	stem	5.8 ± 0.37^h^	1.16 ± 0.53^f^	0.24 ± 0.06^f^	ND	0.16 ± 0.06^e^
	rhizome	19.2 ± 1.42^f^	6.44 ± 0.49^e^	0.79 ± 0.06^d^	1.19 ± 0.26^e^	0.89 ± 0.05^d^
6	leaf	30.4 ± 2.16^c^	9.27 ± 1.03^c^	1.08 ± 0.04^c^	3.66 ± 0.22^b^	1.26 ± 0.31^c^
	stem	5.9 ± 0.33^h^	1.34 ± 0.64^f^	0.20 ± 0.05^f^	ND	0.21 ± 0.05^e^
	rhizome	26.7 ± 2.83^d^	9.86 ± 0.96^c^	1.73 ± 0.53^b^	2.66 ± 0.17^d^	1.73 ± 0.41^b^
9	leaf	22.4 ± 1.69^e^	7.61 ± 0.86^d^	1.05 ± 0.09^c^	3.14 ± 0.19^c^	1.09 ± 0.22^c^
	stem	6.7 ± 0.52^g^	1.18 ± 0.66^f^	0.34 ± 0.03^e^	0.26 ± 0.09^f^	0.20 ± 0.07^e^
	rhizome	44.8 ± 3.16^a^	19.76 ± 2.54^a^	2.71 ± 0.79^a^	3.73 ± 0.36^b^	2.61 ± 0.34^a^

Data are means of triplicate measurements ± standard deviation. Means not sharing a common single letter in each column for each measurement were significantly different at *P* ≤ 0.05. The units of total phenolics and phenolic compounds are mg gallic acid equivalents/g DM and mg/g DM, respectively. ND: not detected

### The Enzyme Chalcone Synthase (CHS, EC 2.3.1.74) Activity

**Fig. 1:**
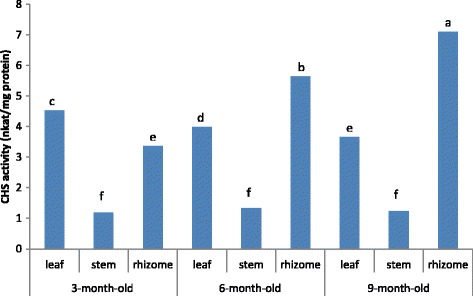
CHS activity in *Z. zerumbet* extracts at different plant age. Means not sharing a common single letter were significantly different at *P*  ≤ 0.05

Enzyme chalcone synthase (CHS, EC 2.3.1.74) has been discovered and reported as a key enzyme for the metabolism of flavonoid in plant cells [31]. Varied CHS activity was observed in *Z. zerumbet* at different growth stages (Fig. 1). CHS activity in the leaf samples decreased significantly as the plant matured from 3 to 9 months (from 4.76 to 3.72 nkat/mg protein) and this reduction was approximately 13.6 % between 3 and 6 months and 9.4 % between 6 and 9 months. CHS activity in the stem enhanced approximately 11.7 % between 3 and 6 months and after that, decreased approximately 6.7 % between 6 and 9 months; however, this alteration in CHS activity did not reach statistical significance. A marked increased in CHS enzyme activity was observed in the rhizomes. CHS activity increased significantly (119.3 %) from 3 to 6 months and approximately 34.3 % increase between 6 and 9 months. Flavonoids are derived from 4-coumaroyl-CoA and malonyl-CoA in the presence of CHS. This indicates that CHS is an important enzyme in flavonoid synthesis. According to the current results, it is hypothesized that the increment of polyphenolic compounds in the 9-month-old rhizomes and 3-month-old leaves could be attributed to an increase in CHS activity. Furthermore, it was reported that CHS could be considered as a biochemical marker for evaluating the dynamic changes in flavonoid synthesis in plants [32].

### Antioxidant activity

Table 3, shows the antioxidant activity of the leaf, stem, and rhizome extracts of *Z. zerumbet* at three different growth stages, using a DPPH and FRAP assay. The antioxidant activity of *Z. zerumbet* was significantly affected by the age of the plant and parts of the plant (Table 3). At concentration of 100 μg/mL, the highest DPPH activity was observed with the 9-month-old rhizome (76.42 %), followed by the 6-month-old rhizome (59.41 %) and 3-month-old leaf (57.82 %), with IC_50_ (the half maximal inhibitory concentration) of 55.8, 86.4, and 98.5 μg/mL, respectively, compared to α-tocopherol (84.19 %, 44.8 μg/mL) and BHT (70.25 %, 58.6 μg/mL). A lower IC_50_ indicates a stronger free radical inhibition (strong free radical inhibitors are active at low concentrations). Interestingly, DPPH activity of the 9-month-old rhizomes was higher than that observed with BHT (positive control). In this study, IC_50_ was not observed in the stem extracts, which suggests poor antioxidant activity in the stem. The FRAP value of plant extracts was 113.6–581.8 μM of Fe (II)/g and the highest FRAP activity was observed in the 9-month-old rhizome, with IC_50_ of 62.4 μg/mL. Similar to the results of the DPPH assay, IC_50_ of the FRAP assay was not observed in the stem extracts. In the leaf extracts, FRAP activity between 3 and 6 months decreased approximately 10.6 %, and 13.7 % between 6 and 9 months. In the rhizomes extracts, FRAP activity increased (approximately 68.8 %) from 3 to 6 months and from 6 to 9 months (approximately 35.6 %). The reduction in antioxidant activity in the leaf and enhancement in the rhizomes could be attributed to the changes in the levels of phytochemicals, such as flavonoids and phenolic acids. Previous studies have reported that there was no significant correlation between phenolic compounds and antioxidant activity [33, 34]. However, the results of some studies demonstrated that the antioxidant activity of herbs was positively correlated and with the levels of flavonoids and phenolic acids [35–37]. Our findings in the current study, corroborate with the results of Yuting et al. [33], who suggested that levels of flavonoid and phenolics corresponds to the free radical scavenging ability of the herbs. Based on our results, 3 and 9 months after plantation are the most suitable harvesting time for the leaf and rhizome, respectively, to obtain *Z. zerumbet* of high pharmaceutical quality. To the best of our knowledge, this is the first study that reported the alteration in the pharmaceutical quality of *Z. zerumbet* at different plant growth stages; thus, the results of the current study could be useful for future studies.

**Table 3:** DPPH and FRAP scavenging activities (at concentration of 100 μg/mL) and IC_50_ value of *Z. zerumbet* extracts

Plant age (month-old)/positive controls	Plant parts	DPPH free radical scavenging activity (%)	IC_50_ (μg/mL)	Ferric reducing antioxidant potential (μM of Fe (II)/g)	IC_50_ (μg/mL)
3	leaf	57.82 ± 3.29^d^	98.5 ± 4.35^d^	489.4 ± 20.16^d^	119.42 ± 4.55^d^
	stem	15.46 ± 1.15^h^	NO	117.7 ± 6.53^i^	NO
	rhizome	34.76 ± 3.31^g^	110.9 ± 3.67^c^	254.0 ± 16.42^g^	134.8 ± 5.21^c^
6	leaf	51.66 ± 3.55^e^	121.4 ± 5.16^b^	437.1 ± 18.73^e^	144.6 ± 4.19^b^
	stem	16.37 ± 1.12^h^	NO	113.6 ± 5.68^i^	NO
	rhizome	59.41 ± 3.68^d^	86.4 ± 4.19^e^	429.0 ± 19.42^e^	99.7 ± 3.55^e^
9	leaf	43.18 ± 2.49^f^	149.7 ± 5.48^a^	377.2 ± 15.27^f^	164.1 ± 4.79^a^
	stem	16.48 ± 1.74^h^	NO	127.2 ± 6.46^h^	NO
	rhizome	76.42 ± 3.29^b^	55.8 ± 4.26^f^	581.8 ± 22.18^b^	62.4 ± 3.28^g^
α-tocopherol		84.19 ± 4.68^a^	44.8 ± 4.33^g^	849.4 ± 24.16^a^	49.1 ± 2.77^h^
BHT		70.25 ± 2.69^c^	58.6 ± 4.75^f^	512.1 ± 17.54^c^	67.7 ± 3.86^f^

Data are means of triplicate measurements ± standard deviation. Means not sharing a common single letter in each column for each measurement were significantly different at *P* ≤ 0.05. NO: not observed

### Antibacterial activity

The antibacterial activity of *Z. zerumbet* extracts from the leaf, stem, and rhizome against both Gram-positive and Gram-negative bacteria is shown in Table 4. Three-month-old leaves, 9-month-old rhizomes, and stems were chosen for investigation of antibacterial activity, since they showed the highest concentration of secondary metabolites and highest antioxidant activity (DPPH and FRAP activity). Leaf and rhizome extracts of *Z. zerumbet* demonstrated good antibacterial activity against Gram-positive and Gram-negative bacteria strains. Rhizomes extract of *Z. zerumbet* showed potent antibacterial activity than that of the leaf extracts, with the exception of *Staphylococcus aureus* (Table 4). Antibacterial activities of the rhizome extract of *Z. zerumbet* against *S. aureus* (7.3 mm), *Bacillus subtilis* (5.9 mm), and *Pseudomonas aeruginosa* (7.0 mm) were higher than those observed with gentamicin (*S. aureus* 6.5 mm, *B. subtilis* 5.6 mm, and *P. aeruginosa* 6.2 mm), and ciprofloxacin ( *B. subtilis* 4.8 mm, and *P. aeruginosa* 6.7 mm). The best antibacterial activity of the leaf extracts was against *S. aureus* (8.7 mm). The reason for higher sensitivity of the Gram-positive bacteria than Gram negative bacteria could be attributed to their differences in cell membrane constituents. The outer membrane found in the Gram-negative cell wall is composed of structural lipopolysaccharides which render the cell wall impermeable to lipophilic solutes, unlike Gram-positive bacteria which do not have this outer membrane. This morphologic difference influences their reaction to antibacterial agents. Stem extract of *Z. zerumbet* exhibited weak antibacterial activity compared to those of the leaf and rhizome extracts. In addition, stem extract of *Z. zerumbet* did not show antibacterial activity against *Listeria monocytogenes*, *Escherichia coli*, *Salmonella typhimurium*, and *P. aeruginosa* bacterial strains.

**Table 4:** Antibacterial activity of *Z.zerumbet* extracts (three month leaf and nine month rhizome and stem) and antibiotics against bacterial strains

Bacterial Strains		Inhibition zone (mm)				
	Leaf	Stem	Rhizome	Gentamicin	Ciprofloxacin	DMSO:water (1:9 v/v)
*Staphylococcus aureus*	8.7 ± 0.291^a^	1.4 ± 0.173^d^	7.3 ± 0.337^b^	6.5 ± 0.277^c^	7.3 ± 0.276^b^	NO
*Bacillus subtilis*	5.1 ± 0.188^c^	1.2 ± 0.229^e^	5.9 ± 0.121^a^	5.6 ± 0.114^b^	4.8 ± 0.129^d^	NO
*Listeria monocytogenes*	1.0 ± 0.164^d^	NO	1.8 ± 0.367^c^	4.0 ± 0.127^ab^	4.2 ± 0.119^a^	NO
*Escherichia coli*	1.5 ± 0.146^c^	NO	3.5 ± 0.322^b^	5.4 ± 0.318^a^	5.5 ± 0.337^a^	NO
*Salmonella typhimurium*	4.1 ± 0.267^d^	NO	5.3 ± 0.379^c^	7.2 ± 0.272^a^	6.8 ± 0.252^b^	NO
*Pseudomonas aeruginosa*	5.6 ± 0.218^c^	NO	7.0 ± 0.381^a^	6.2 ± 0.365^b^	6.7 ± 0.322^a^	NO

All analyses are the mean of triplicate measurements ± standard deviation. Means not sharing a common letter in each row were significantly different at *P* ≤ 0.05; NO: not observed

Minimal Inhibitory Concentration (MIC) of *Z. zerumbet* extracts ranged from 30 to 100 μg/mL (Table 5). A lower MIC value indicates a stronger antibacterial activity (strong bacterial inhibitors are active at low concentrations). Therefore, the results indicated that among the investigated bacteria strains, *S. aureus* was sensitive to the leaf extract of *Z. zerumbet,* with MIC of 30.0 μg/mL and others were sensitive to the rhizome extracts, with MIC of 40–100 μg/mL. MIC of the positive controls (gentamicin and ciprofloxacin) ranged from 0.20 to 1.00 μg/mL, which were lower than that of *Z. zerumbet* extracts.

**Table 5:** Minimal Inhibitory Concentration (MIC) of *Z.zerumbet* extracts (3 month-old leaf and 9 month-old rhizome and stem) against bacterial strains

Bacterial Strains	Leaf	Stem	Rhizome
*Staphylococcus aureus*	30.0	>100	40.0
*Bacillus subtilis*	80.0	>100	60.0
*Listeria monocytogenes*	>100	NO	>100
*Escherichia coli*	>100	NO	>100
*Salmonella typhimurium*	>100	NO	50.0
*Pseudomonas aeruginosa*	80.0	NO	40.0

All analyses are the mean of triplicate measurements ± standard deviation; unit is μg/mL; NO: not observed

The rhizome extract of *Z. zerumbet* have previously demonstrated antibacterial activity against *E. coli, P. aeruginosa, Sarcina lutea,* and *B. cereus,* with MIC of 128, 128, 128, and 256 μg/mL, respectively [16]. A study reported that the oil of *Z. zerumbet* rhizome showed significant inhibitory activity against bacteria strains, *Lactococcus lactis* (80 mm), and *S. aureus* (120 mm) and fungus, *Fusarium oxysporum* (100 mm) and *Aspergillus awomori* (150 mm) [38] using the disc agar diffusion method. The major limitation of previous studies was that the age of *Z. zerumbet* harvested was not reported when antibacterial activity, antioxidant activity, and phytochemicals were analyzed. This should be noted in future studies. These findings may help us to determine the optimum time of harvest for *Z. zerumbet*. It has been surmised that the antibacterial activities of herbs are focused on the structures and cellular membranes and due to the presence of various bioactive compounds and extensive phytochemical profiles, it is likely that the antimicrobial potency is not just caused by one solitary mechanism but rather by several events at a cellular level [39].

## Conclusion

The purpose of the current study was to determine the changes in phytochemical synthesis and pharmaceutical quality of *Z. zerumbet* extracts at different growth stages. This study has shown that during the plant maturity between 3 and 9 months, the production of flavonoids and phenolic acids decreased in the leaf, and increased in the rhizomes. Among the flavonoids and phenolic acids identified in this study, catechin, quercetin, gallic acid, and ferulic acid were present in large numbers. The results of the antioxidant activity indicated that the parts of plants with high concentrations of flavonoids and phenolic acids demonstrated the highest DPPH and FRAP activity. Leaf and rhizome extracts of *Z. zerumbet* showed antibacterial activity against both Gram-positive and Gram-negative bacteria strains. This study suggests that the optimum time for harvesting the leaves of *Z. zerumbet* is 3 months after planting and for rhizomes is 9 months after plantation, to ensure a high level of secondary metabolites. This study has indicated the importance of the age of the plant for the accumulation of secondary metabolites and pharmaceutical quality in *Z. zerumbet*.
